# Prevalence and risk factors of hepatitis B and C virus infections in an impoverished urban community in Dhaka, Bangladesh

**DOI:** 10.1186/1471-2334-10-208

**Published:** 2010-07-15

**Authors:** Hasan Ashraf, Nur H Alam, Christian Rothermundt, Abdullah Brooks, Pradip Bardhan, Lokman Hossain, Mohammed A Salam, Mohammed S Hassan, Christoph Beglinger, Niklaus Gyr

**Affiliations:** 1 ICDDR,B (International Centre for Diarrhoeal Disease Research, Bangladesh), 68 Shaheed Tajuddin Ahmed Sarani, Mohakhali, Dhaka 1212, Bangladesh; 2 University of Basel, Petersgraben 4, CH-4031, Basel, Switzerland; 3 Johns Hopkins Bloomberg School of Public Health, 615 N. Wolfe Street, Baltimore, MD 21205, USA; 4 BIRDEM (Diabetic Hospital), 122, Kazi Nazrul Islam Avenue, Dhaka 1000, Bangladesh

## Abstract

**Background:**

Viral hepatitis is a serious global public health problem affecting billions of people globally, and both hepatitis B virus (HBV) and hepatitis C virus (HCV) infections are rapidly spreading in the developing countries including Bangladesh due to the lack of health education, poverty, illiteracy and lack of hepatitis B vaccination. Also there is lack of information on their prevalence among the general population. So, a population-based serological survey was conducted in Dhaka to determine the prevalence and risk factors of HBV and HCV infections.

**Methods:**

Healthy individuals were selected for demographic and behavioural characteristics by stratified cluster sampling and blood tested for hepatitis B surface antigen (HBsAg), antibody to HBV core antigen (anti-HBc), and anti-HCV antibodies (anti-HCV).

**Results:**

From June 2005-November 2006, 1997 participants were screened for HBsAg, anti-HBc and anti-HCV, 738 (37%) were males with mean (SD) age of 24 (14) years. HBV-seropositivity was documented in 582 (29%) participants: 14 (0.7%) were positive for HBsAg, 452 (22.6%) for anti-HBc and 116 (5.8%) for both HBsAg and anti-HBc. Four (0.2%) participants were positive for anti-HCV, and another five (0.3%) for both anti-HBc and anti-HCV. Ninety-six/246 (39%) family members residing at same households with HBsAg positive participants were also HBV-seropositive [74 (30.1%) for anti-HBc and 22 (8.9%) for both HBsAg and anti-HBc], which was significantly higher among family members (39%) than that of study participants (29%) (OR 1.56; p < 0.001). In bivariate analysis, HBV-seropositivity was significantly associated with married status (OR 2.27; p < 0.001), history of jaundice (OR 1.35; p = 0.009), surgical operations (OR 1.26; p = 0.04), needle-stick injuries (OR 2.09; p = 0.002), visiting unregistered health-care providers (OR 1.40; p = 0.008), receiving treatment for sexually transmitted diseases (STD) (OR 1.79; p = 0.001), animal bites (OR 1.73; p < 0.001); ear-nose-body piercing in females (OR 4.97; p < 0.001); circumcision (OR 3.21; p < 0.001), and visiting community barber for shaving in males (OR 3.77; p < 0.001). In logistic regression analysis, married status (OR 1.32; p = 0.04), surgical operations (OR 1.39; p = 0.02), animal bites (OR 1.43; p = 0.02), visiting unregistered health-care providers (OR 1.40; p = 0.01); and ear-nose-body piercing in females (OR 4.97; p < 0.001) were significantly associated with HBV-seropositivity.

**Conclusions:**

The results indicate intermediate level of endemicity of HBV infection in Dhaka community, with much higher prevalence among family members of HBsAg positive individuals but low prevalence of HCV infections, clearly indicating need for universal hepatitis B vaccination. The use of disposable needles for ear-nose-body piercing need to be promoted through public awareness programmes as a preventive strategy.

## Background

Viral hepatitis is a serious public health problem affecting billions of people globally. Caused mainly by hepatitis viruses A, B, C, D and E, and rarely by cytomegalovirus (CMV), Epstein-Barr virus (EBV) and fungal infections, the spectrum of hepatitis range from sub-clinical to milder and life threatening illness including hepatocellular carcinoma [1,2]. Globally two billion people are infected with HBV, and 350 millions of them have chronic (lifelong) infections, who are at high risk of death from liver cirrhosis and liver cancer that kill more than one million people globally each year [3]. In the Middle East and Indian sub-continent, HBV infection is of intermediate endemicity with chronic HBV carriage rate of 2-5% among general population [3]. In Bangladesh, there is paucity of information on the prevalence of HBV infections among general population and majority of the previous studies were conducted in selected group of people with higher risk factors such as blood donors, drug addicts, commercial sex workers (CSWs) or hospitalised patients [4-8]. However, a recent report showed 5.5% HBsAg positivity among the general population living in Savar, a semi-urban area on the outskirts of Dhaka [9]. Although HBsAg is the most reliable biological biomarker of HBV infection, and the anti-HBc antibody is an important marker for surveying the burden of HBV infection as it persists even after resolution of infection, and thus identifies both past and current HBV infection [10]. As majority of the previous studies in Bangladesh examined only the prevalence of HBsAg [2,4-7,11-16] and most of them were conducted in selected group of people with higher risk factors, we decided to estimate the prevalence of both HBsAg and anti-HBc among the general population of Dhaka, Bangladesh.

HCV infections is also a major global health problem with an estimated 170 million people chronically infected and 3-4 million people get new infections each year [17]. HCV infections lead to acute hepatitis in 20% cases, and chronic hepatitis in 50-80% cases, of whom 10-20% develop liver cirrhosis [17-20] and 1-5% develop liver cancer in 20-30 years [17]. The prevalence of HCV infection varies throughout the world with significant regional and ethnic differences. The highest number reported from Egypt following parenteral antischistosomal therapy with a prevalence of anti-HCV of 6-28% (mean 22%) depending on the regions [21]. Although some risk factors for acquiring HCV infections are present in 50% cases, no recognizable transmission factor could be identified in the remaining 50% [17,22]. As there is also lack of information on the prevalence of HCV infection among the general population and nearly all previous studies were conducted in selected group of people [8,23-25], we also decided to estimate the prevalence of anti-HCV in the same population.

It is evident that HBV and HCV infections are both major global health problems, and they are rapidly spreading in developing countries due to the lack of health education, poverty, illiteracy and lack of hepatitis B vaccination. As many chronically infected individuals remain asymptomatic, and thus undetected for many years, we planned this population-based serological study to determine the prevalence of HBsAg, anti-HBc, and anti-HCV among all age groups and to identify the possible risk factors for acquiring the infections. We hoped that the findings might guide eventually the development, adaptation, and evaluation of prevention strategies.

## Methods

### Study site

The study was conducted in Kamalapur, a densely populated community in urban Dhaka, the capital city of Bangladesh. The International Centre for Diarrhoeal Disease Research, Bangladesh (ICDDR, B) has been using this as its first urban field site since 1998. It is an impoverished area comprised of seven communities in four municipal wards with 200,000 residents in an area of 4 km^2 ^(29,663 persons/km^2^), 11.2% of whom are under-5 children, with a mean (range) house hold size of 4 (1 to 20) persons. They have a median monthly household income of US $50. Kamalapur is divided into seven geographical strata and 450 clusters, each with approximately 100 households. The community had all the potential reported risk factors, such as overcrowding, low income, poor sanitation for high disease burden of infectious diseases such as diarrhea, and pneumonia [26,27] and hepatitis.

### Sample size

Assuming that 6% of the population will have a HBsAg positive status with 1% precision and 95% confidence limit, we estimated the sample size to be 1955. For HCV, we assumed that 5% of the population will have an anti-HCV positive status with 1% precision and 95% confidence limit, we estimated the sample size to be 1825. Assuming a refusal rate of 10%, we determined the sample size to be 2000. To get sufficient number of people to detect the above mentioned prevalence, we considered Kamalapur, Dhaka, as the population suitable for this study.

### Randomization

A population based, cross-sectional survey was conducted in 2000 individuals, aged 0-60 years during 2005-2006. For achieving this sample, all the 450 geographical clusters of Kamalapur were selected using random sampling. The first household of the cluster was chosen randomly and if a locked house was encountered, then the next household in same direction replaced that.

### Study procedure

The trained field research assistants (FRAs) visited the selected households in accordance with the randomisation list and approached the head of the family. They explained the purpose and objective of the study and obtained written informed consent from study participants, or parents for eligible children. They administered a pre-tested questionnaire to the participants at a mutually agreed date at the clinic, when a research physician collected 4 ml of blood from the antecubital veins under aseptic conditions. The blood specimens were centrifuged at the clinic within 6 hours and separated sera were transported to the Dhaka Hospital of ICDDR, B, and stored at -20°C in aliquots. For diagnosis of HBV infection, HBsAg and anti-HBc were determined using commercially available enzyme-linked immunosorbent assay (ELISA) kits (Manufacturer: DiaSorin S. A., Italy). For diagnosis of HCV infection, anti-HCV antibody was detected using a third-generation ELISA kit (Manufacturer: DiaSorin S. A., Italy). After collection of blood samples, the research physician interviewed the participants (parents if the participant was a minor) in a private set up within the clinic to collect their socio-demographic characteristics including age, gender, years of education, socio-economic status, occupation, income, and marital status by using a structured questionnaire. They were also asked about the type of health care providers they consulted for health problems, history of jaundice, history of taking injections, previous surgical procedures, frequency of dental visits, receiving blood or blood products, history of current/past use of intravenous drugs, siblings, history of tattooing; ear-nose-body piercing in females, and circumcision and visiting community barbers for shaving in males. Some questions about sexual behaviour such as having multiple sexual partners, having received treatment for STD were asked to participants 18 years of age or older by research physician of the same sex. The study was approved by the Research and Ethical Review Committees of ICDDR, B.

The laboratory test results were kept confidential and the research physician shared the results with the participants. Infected individuals were provided with appropriate information on the prevention of spread of these infections to others, and referred them to the nearest public health care facilities.

### Serological tests

We only performed HBsAg, anti-HBc, and anti-HCV to reduce the laboratory test costs.

#### HBsAg

is the serologic hallmark of HBV infection. It appears in serum 1-10 weeks after an acute exposure to HBV, prior to the onset of hepatitic symptoms. The sensitivity of HBsAg is 100%, the specificity is 99.7%, and the limit of detection is 0.05 PEI units/ml. The lower limit of detection, PEI unit was explained as the analytical sensitivity that may be expressed as the limit of detection, which is the minimal amount of specific analyte precisely detectable by the assay. A conversion of 0.05 PEI units/ml to international unit (IU) values was evaluated by testing HBsAg international reference material from World Health Organization NIBSC 1^st ^International Standard, code 80/549; (HBsAg, subtype *ad*) and limit of detection was found to be 0.05 IU/ml. When the same protocol was used with Paul-Ehrlich-Institute (Germany), HBsAg reference preparation (subtypes *ad*, *ay*), the detection limit came to 0.03 PEI units/ml. If the infection is self-limited, HBsAg disappears in most patients before the serum hepatitis B surface antibody (anti-HBs) can be detected. Persistence of HBsAg for more than six months implies chronic infection.

#### Anti-HBc

identifies individuals with both current and past HBV infection (prevalence). The sensitivity of anti-HBc is 100%, the specificity is 99.83%, and the limit of detection is < 0.5 PEI units/ml. Almost all HBV-infected people usually develop anti-HBc. Detection of anti-HBc indicates exposure to HBV, which may be acute, chronic, or resolved infection. Fourteen (0.7%) study subjects were expressing HBsAg positive, but negative for anti-HBc. Although rare, this serological profile of HBsAg positive, but anti-HBc negative is not that exceptional. In addition to the diagnostic kits efficacy, the immunosuppressive state of the subject may also contribute to such profile [28].

#### Anti-HCV

The discovery of HCV in 1989 led to the development of an antibody diagnostic assay (anti-HCV) based on viral recombinant peptides. The third generation assays (ELISA-3) have been introduced incorporating antigens from putative neucleocapsid, NS3, NS4, and NS5 regions, and become positive in 2-3 weeks after the infection [29]. They are currently the most widely used screening tests for HCV and are more sensitive and specific than earlier generation tests in screening blood donors [30]. Detection of anti-HCV indicates present or previous HCV infection but cannot discriminate acute from chronic or resolved HCV infection [31].

### Statistical Analysis

All data were analysed using Statistical Package for Social Sciences (SPSS) version 10.0 [32]. The analysis was carried out at three levels of descriptive, bivariate and logistic regression analysis. Descriptive statistics of socio-demographic variables and other characteristics of the sampled population were computed. Means and SD were calculated for quantitative variables and proportions for categorical variables. Percentage with 95% confidence interval (CI) was used to describe the prevalence. OR and 95% CI was calculated for each association. Associations among independent variables were assessed using appropriate tests, such as *x*^2 ^or Fisher's exact tests, when indicated before performing logistic regression analysis. Multiple logistic regression models were used to examine the association between independent variables and the main outcome variable, HBV-seropositivity, while controlling for the effects of other covariates. All variables which were associated with outcome in bivariate analysis were included in the model. In logistic model, reference category (OR = 1) for OR estimate was HBV-negative participants. A probability of < 0.05 was considered as statistical significant. The final logistic regression model was obtained by forward selection based on single variables and their possible interactions. Given small number of hepatitis C infections, no tests of association could be performed.

## Results

**Figure 1:**
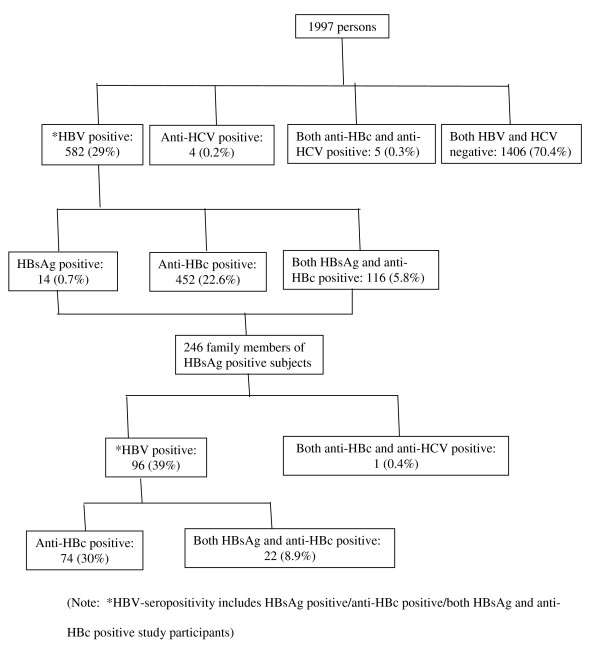
Study Profile

From June 2005 to November 2006, 2004 subjects were initially selected for the study, of whom 1997 participants were enrolled into the study, mostly young adults, 738 (37%) were males and 1259 (63%) were females, with a mean (SD) age of 24 (14) years. Seven subjects were not enrolled as we failed to confirm the serological diagnosis. The evidence for current or previous HBV infection was documented in 582 (29%) participants with a male to female ratio of 40:60 and a mean (SD) age of 28 (13) years. HBsAg was positive in 14 (0.7%) participants, anti-HBc was positive in 452 (22.6%) participants, and both HBsAg and anti-HBc were positive in 116 (5.8%) participants (Table 1). Four (0.2%) participants were positive for anti-HCV, and another five (0.3%) for both anti-HBc and anti-HCV (Table 1). The HBV positivity was significantly more among females than male participants (40% vs. 10%) (OR 5.75; p < 0.001). The HBV positivity among under-5 children were 60/208 (28.8%): HBsAg was positive in 5/208 (2.4%) children, anti-HBc was positive in 34/208 (16.3%), and both HBsAg and anti-HBc were positive in 21/208 (10%) children (Table 1). The anti-HBc positivity was significantly less among under-5 children than that of study participants (16.3% vs. 22.6%) (OR 1.47; p < 0.01). After receiving laboratory results, 246 family members residing at the same household as that of the study participants' positive for HBsAg were similarly studied. In total, 96/246 (39%) of them were HBV-seropositive with a male to female ratio of 45:55 and a mean (SD) age of 21 (16) years: 74 (30.1%) were positive for anti-HBc, and 22 (8.9%) were positive for both HBsAg and anti-HBc (Figure 1). In fact, the prevalence of HBV infection among the family members (96/246; 39%) was significantly higher than that of the study participants (582/1997; 29%) (OR 1.56; p < 0.001). Hepatitis B vaccination was found to be protective against HBV infection [only 10/582 (1.7%) of the HBV positive participants were vaccinated in contrast to 66/1406 (4.7%) of the HBV negative participants were vaccinated] (OR 2.82; p = 0.001).

**Table 1:** Groups of study participants and family members based on the serological test results

Positive test results	Study participants (n = 1997)	Under-5 children (n = 208)	Family members (n = 246)	Interpretation
1. HBsAg	14 (0.7%)	5 (2.4%)	0	Acute or chronic HBV infection

2. Anti-HBc	452 (22.6%)	34 (16.3%)	74 (30%)	Exposure to HBV, which may be acute, chronic or resolved infection

3. Both HBsAg and anti-HBc	116 (5.8%)	21 (10%)	22 (8.9%)	Acute or chronic HBV infection

4. Anti-HCV	4 (0.2%)	0	0	Present or previous HCV infection

5. Both anti-HBc and anti-HCV	5 (0.3%)	0	1 (0.4%)	Mixed HBV and HCV infections

### Risk factors for HBV-seropositivity

In bivariate analysis, HBV-seropositivity (including HBsAg positive, or anti-HBc positive, or both HBsAg and anti-HBc positive participants) was significantly associated with a variety of sociodemographic and behavioral independent variables, such as married status (OR 2.27; p < 0.001), history of jaundice (OR 1.35; p = 0.009), surgical operations (OR 1.26; p = 0.04), needle-stick injuries (OR 2.09; p = 0.002), visiting unregistered health-care providers (OR 1.40; p = 0.008), receiving treatment for STD (OR 1.79; p = 0.001), animal bites (OR 1.73; p < 0.001); ear-nose-body piercing in females (OR 4.97; p < 0.001); circumcision (OR 3.21; p < 0.001) and visiting community barber for shaving in males (OR 3.77; p < 0.001) (Table 2).

**Table 2:** Bivariate analysis for significant risk factors for *HBV-seropositivity

Risk factors	*HBV positive n = 582 (100%)	HBV negative n = 1406 (100%)	Odds Ratio (95% CI)	*p*-value
1. Marital status				

Un-married	200 (34.4)	764 (54.3)	1.0	

Married	382 (65.6)	642 (45.7)	2.27 (1.85-2.79)	< 0.001

2. History of jaundice				

No	431 (74.1)	1116 (79.4)	1.0	

Yes	151 (25.9)	290 (20.6)	1.35 (1.07-1.70)	0.009

3. Previous surgery				

No	431 (74.1)	1101 (78.3)	1.0	

Yes	151 (25.9)	305 (21.7)	1.26 (1.00-1.59)	0.04

4. Circumcision in males only (n = 734)				

No	37 (15.9)	190 (37.8)	1.0	

Yes	195 (84.1)	312 (62.2)	3.21 (2.13-4.86)	< 0.001

5. Needle-stick injuries				

No	550 (94.5)	1368 (97.3)	1.0	

Yes	32 (5.5)	38 (2.7)	2.09 (1.26-3.47)	0.002

6. Ear-nose-body piercing in females only (n = 1254)				

No	14 (4)	155 (17.1)	1.0	

Yes	336 (96)	749 (82.9)	4.97 (2.76-9.10)	< 0.001

7. Animal bites				

No	451 (77.5)	1204 (85.6)	1.0	

Yes	131 (22.5)	202 (14.4)	1.73 (1.34-2.23)	< 0.001

8. Visiting community barber for shaving in males only (n = 734)				

No	64 (27.6)	296 (59)	1.0	

Yes	168 (72.4)	206 (14)	3.77 (2.65-5.37)	< 0.001

9. Visiting unregistered health care providers				

No	98 (16.8)	310 (22.0)	1.0	

Yes	484 (83.2)	1096 (78.0)	1.40 (1.08-1.81)	0.008

10. Receiving treatment for STD				

No	528 (90.7)	1330 (94.6)	1.0	

Yes	54 (9.3)	76 (5.4)	1.79 (1.23-2.61)	0.001


(Note: *HBV-seropositivity includes HBsAg positive/anti-HBc positive/both HBsAg and anti-HBc positive study participants)

After adjusting for potential confounding effects in the logistic regression analysis, married status (OR 1.32; p = 0.04), surgical operations (OR 1.39; p = 0.02), animal bites (OR 1.43; p = 0.02), visiting unregistered health-care providers (OR 1.40; p = 0.01); and ear-nose-body piercing in females (OR 4.97; p < 0.001) remained independent determinants of HBV-seropositivity (Table 3). In the above regression model, both age and sex were retained as covariates to control their influence on other covariates.

**Table 3:** Multiple logistic regression analysis for being *HBV-seropositive

Risk factors	*HBV positive n = 582 (100%)	HBV negative n = 1406 (100%)	Adjusted Odds Ratio (95% CI)	*p*-value
1. Married status	382 (65.6)	642 (45.7)	1.32 (1.00-1.73)	0.046

2. Previous surgery	151 (25.9)	305 (21.7)	1.39 (1.05-1.82)	0.02

3. Ear-nose-body piercing in females (n = 1254)	336/1085 (31)	14/169 (8.3)	4.97 (2.76-9.10)	< 0.001

4. Animal bites	131 (22.5)	202 (14.4)	1.43 (1.04-1.96)	0.024

5. Visiting unregistered health-care providers	484 (83.2)	1096 (78.0)	1.40 (1.08-1.82)	0.012

(Note: *HBV-seropositivity includes HBsAg positive/anti-HBc positive/both HBsAg and anti-HBc positive study participants.

Initial models included the variables age, sex, married status, history of jaundice, surgical operation, circumcision, needle-stick injuries, animal bites, visiting unregistered health care providers, receiving treatment for STD, ear-nose-body piercing in females, circumcision, and visiting community barber for shaving in males. Significant factors were retained in the final model)

## Discussion

Although prevalence studies are not always easily undertaken in the developing countries such as Bangladesh due to high cost, we made efforts to prospectively estimate the prevalence of HBV and HCV infections among a population living at Kamalapur, a densely populated community in Dhaka, the capital city of Bangladesh.

The results of our study suggest a high HBV exposure among our study population. The HBsAg prevalence of 6.5% among our study population is within the range of 2-7%, reported by previous studies from selective population of Dhaka: 3% among healthy adults and children [33] and 3.5% among pregnant women [5]; however, much lower rate (0.8%) was observed among school children [16]. The prevalence was also within the same range as reported from some high-risk groups from Dhaka: 4.4% among non-injectable drug users (non-IDUs) and 6.2% in injectable drug users (IDUs) [24]; and 5.9% among truck drivers and helpers [34]. The higher rates among our study population could be attributed to the general lack of proper health care because of deprived socio-economic status (monthly household income of US $50) and less public health awareness about the transmission of HBV infection as well as the lack of hepatitis B vaccination in the community. Our prevalence was similar (6.5% vs. 6.4%) to that reported from the Rangpur district of Bangladesh [13]. However, higher prevalence has also been reported among the high-risk groups of Dhaka: 7.6% among women at a STD clinic [23]; 8% among IDUs [6]; 8.6% among surgically operated patients [7]; 9.7% among CSWs [8]; 19% among hepatitis patients [2]; and 47% among hepatocellular carcinoma patients [14].

The prevalence of anti-HBc in our study population was similar (22.6% vs. 21.1%) to that reported from healthy adults and children [33] but lower than that reported from the high-risk groups of Dhaka: 24.1% in non-IDUs and 31.8% in IDUs [24]; 35.2% among women at a STD clinic [23]; 48.1% among truck drivers and helpers [34]; 49.3% among women living near a truck stand [25]; and 73% among CSWs [8].

The 0.2% prevalence of anti-HCV observed in our study population is lower than that reported from high-risk groups of Dhaka: 0.8% among truck drivers and helpers [34]; 0.9% among women at a STD clinic [23]; 1.6% among women living near a truck stand [25]; 5.8% in non-IDUs and 24.8% in IDUs [24]; and 13% among hepatitis patients [2].

The intermediate rate of chronic HBV carriage of around 3% was observed in most general populations (clinics, villagers), suggesting that this population would benefit from universal hepatitis B vaccination [11]. In 2004, the Government of Bangladesh and UNICEF have introduced the hepatitis B vaccine into the Expanded Programme on Immunization (EPI) against six infectious diseases. The successful continuation of the programme is expected to reduce chronic HBV infections in the next generations. Since 90% of the HBV infected older children and adults successfully clear the infection and do not become chronic carriers, the prevalence of HBsAg alone might not describe the total burden of HBV infections. Therefore, estimation of the prevalence of anti-HBc, in addition to the estimation of the prevalence of HBsAg which is the most reliable biological biomarker of HBV infection, is much more informative about indicator of HBV disease burden among the population. This probably accounts for our higher anti-HBc (22.6%) than HBsAg (6.5%) seropositivity rates (Figure 1).

There are some limitations of our study. First, we did not perform some diagnostic tests for HBV, e.g. anti-HBc IgM, the presence of which indicates acute infection; and anti-HBs that differentiates susceptible persons from those immune persons, which can be due either to natural infection or hepatitis B vaccination. Second, we did not perform some diagnostic tests for HCV, e. g. recombinant immunoblot assay (RIBA) to confirm HCV exposure, or polymerase chain reaction (PCR) to detect HCV infected individuals. All the above limitations are mainly due to study cost constraints, mostly related to laboratory tests. The third limitation is that the study was conducted in a single population in Dhaka, and may not reflect all of Bangladesh, although the literature we have cited suggests that it should. The fourth limitation is the selection of Kamalapur as the study site, as the activities of ICDDR,B might influence the prevalence of HBV and HCV, which might be a biased location because the people of this area might have altered KAP (knowledge, attitude and performance) regarding infectious diseases due to prolonged presence of ICDDR,B team and their health education. A final limitation is the relatively short observation window, which may have missed important secular trends in the background prevalence of both the hepatitis B and C viruses.

## Conclusions

The results of our study indicate intermediate level of endemicity of HBV infection such as overall intermediate prevalence of HBsAg; but high anti-HBc, indicating that the members of this urban community are highly exposed to a high prevalence of the hepatitis B virus, which may be acute, chronic or resolved, in an urban community in Dhaka, Bangladesh. We also noted higher prevalence among the family members residing at the same household of that of the HBsAg positive individuals, suggesting likely intra-household transmission. However, we observed a much lower prevalence of HCV infections in the same community. We also identified some independent risk factors, which could help health care providers and policy makers in designing and initiating effective preventive programmes. The findings also highlight the need for prevention and control of HBV infection in Bangladesh by implementing universal hepatitis B vaccination and creating public awareness to promote the use of disposable needles for ear-nose-body piercing.

Future follow-up studies are required to confirm the family clustering effect of HBV infection as observed in Korea [35], and to better define the transmission dynamics, and to identify their common risk factors for acquiring HBV infection by comparing family members of HBsAg positive participants with those who are negative for the tests. Such a study is planned to be conducted for the same community in Dhaka. Further, more long-term population-based surveillance studies, with extended serology of HBV infection, are needed to more accurately assess the hepatitis B true disease burden in Bangladesh, the impact of vaccination, and to guide prioritization of scarce health care resources.

## Abbreviations

ICDDR,B: International Centre for Diarrhoeal Disease Research, Bangladesh; HBV: Hepatitis B virus; HCV: Hepatitis C virus; HBsAg: Hepatitis B surface antigen; Anti-HBc: Antibody to hepatitis B core antigen; Anti-HCV: Anti-HCV antibody; SD: Standard deviation; OR: Odds ratio; STD: Sexually transmitted diseases; CMV: Cytomegalovirus; EBV: Epstein-Barr virus; CSWs: Commercial sex workers; FRAs: Field research assistants; ELISA: Enzyme-linked immunosorbent assay; SPSS: Statistical Package for Social Sciences; CI: Confidence Interval; Anti-HBs: Hepatitis B surface antibody; PEI: Paul-Ehrlich-Institute; Non-IDUs: Non-injectable drug users; IDUs: Injectable drug users; UNICEF: The United Nations International Children's Fund; EPI: Expanded Programme on Immunization; RIBA: Recombinant immunoblot assay; PCR: Polymerase chain reaction; KAP: Knowledge, attitude and performance.

## Competing interests

The authors declare that they have no competing interests.

## Authors' contributions

HA, CR, NG conceived the idea; HA, CR, NG, NHA, LH contributed to the study data interpretation; HA, MAS, NG wrote the paper. HA, CR, NG, NHA, LH, MAS, WAB, PB, MSH, CG critically analyzed and approved the final manuscript.

## Pre-publication history

The pre-publication history for this paper can be accessed here:

http://www.biomedcentral.com/1471-2334/10/208/prepub
